# Growth Factors in Oral Tissue Engineering: New Perspectives and Current Therapeutic Options

**DOI:** 10.1155/2021/8840598

**Published:** 2021-01-06

**Authors:** Luca Fiorillo, Gabriele Cervino, Pablo Galindo-Moreno, Alan Scott Herford, Gianrico Spagnuolo, Marco Cicciù

**Affiliations:** ^1^Department of Biomedical and Dental Sciences, Morphological and Functional Images, University of Messina, Policlinico G. Martino, Via Consolare Valeria, 98100 Me, Italy; ^2^Oral Surgery and Implant Dentistry Department, School of Dentistry, University of Granada, Granada, Spain; ^3^Department of Maxillofacial Surgery, Loma Linda University, Loma Linda, CA 92354, USA; ^4^I. M. Sechenov First Moscow State Medical University, Institute of Dentistry, Moscow, Russia; ^5^Department of Neurosciences, Reproductive and Odontostomatological Sciences, University of Naples “Federico II”, Via Pansini 5, 80131 Naples, Italy

## Abstract

The present investigation is aimed at systematically analyzing the recent literature about the innovative scaffold involved in the reconstructive surgeries by applying growth factors and tissue engineering. An extensive review of the contemporary literature was conducted according to the PRISMA guidelines by accessing the PubMed, Embase, and Scopus Elsevier databases. Authors performed the English language manuscript research published from 2003 to 2020. A total of 13 relevant studies were included in the present review. The present systematic review included only papers with significant results about correlation between scaffold, molecular features of growth factor, and reconstructive surgeries in oral maxillofacial district. The initial research with filters recorded about 1023 published papers. Beyond reading and considering of suitability, only 42 and then 36 full-text papers were recorded for the revision. All the researches recorded the possibility of using growth factors on rebuilding atrophic jaws. Different growth factors like morphogenetic factors, cytokines, and inflammatory ones and their application over different scaffold materials were recorded. Further investigations should be required in order to state scientific evidence about a clear advantage of applying tissue engineering for therapeutic purpose.

## 1. Introduction

### 1.1. Background

In recent years, growth factors have been introduced as a therapeutic option in the treatment of several congenital and acquired craniofacial defects. Specifically, in the last 20 years, there has been expanding involvement in tissue regeneration in the maxillofacial area. Treatment and management of the atrophic jaws by performing reconstructive treatment involving craniofacial region still represent a challenge for clinician and surgeons. Set aside the first encouraging in vitro results supported by several clinical outcomes, the international scientific community is still having not defined guidelines, but only “suggestions or recommendations” detailing indications and predictable field of applications, for usage of growth factor scaffold [1, 2]. In biology, the term growth factor refers to proteins capable of stimulating cell proliferation, differentiation, and preventing apoptosis [3]. They are typical signal molecules used for communication between the cells of an organism; for example, cytokines (inflammatory molecules) or hormones that bind to specific receptors on the cell membrane of their targets. The main function of growth factors is the external control of the cell cycle, through the abandonment of cellular quiescence (phase G0) and the entry of the cell into phase G1 (of growth). But this is not their only function; in fact, they regulate the entry into mitosis, cell survival, migration, and cell differentiation [3]. Together with proliferation, they always promote differentiation and maturation at the same time (in fact, a proliferation without differentiation means the onset of a tumor). These effects are the most disparate according to the factor; for example, the bone morphogenetic protein (BMP) stimulates the differentiation of osteoblasts, while the vascular endothelial growth factor (VEGF) stimulates the growth of the vessels. The transforming growth factor beta (or TGF-*β*) is a secreted protein (therefore present in the extracellular space) which is part of the group of cytokines. It exists in at least three isoforms called TGF-*β*1, TGF-*β*2, and TGF-*β*3. Often for TGF-*β*, it refers to TGF-*β*1, which was the first discovered member of this protein family. The TGF-*β* protein family is part of the transforming growth factor beta superfamily, which includes activins, inhibins, anti-Mullerian hormone, bone morphogenetic protein, decapentaplegic, and Vg-1. Its receptor has kinase activity in serine threonine. The roles played by TGF-*β* signaling include controlling proliferation and differentiation in most cells. It plays a role in immunity, cancer, bronchial asthma, heart disease, diabetes mellitus, Loeys-Dietz syndrome, Parkinson's disease, and AIDS [4, 5]. TGF-*β* overexpression is responsible for Marfan syndrome [6], an autosomal dominant disorder that primarily affects connective tissue. It also appears to have a role in reproductive function, development, motility, adhesion, bone morphogenesis, and wound healing. This role is diversified according to the tissues in which they are secreted and the quantities in which they are expressed; in some cases, they can also act as potent growth inhibitors as has been observed in a variety of epithelial, endothelial, and lymphoid cells [7–11].

Most tissues have a high expression of TGF-*β*-coding genes. This contrasts with other anti-inflammatory cytokines such as interleukin 10, whose expression is minimal in unstimulated tissues and appears to be required by the pathogenic or commensal bacterial flora. TGF-beta acts as an antiproliferative factor in epithelial cells in the early stages of oncogenesis. Some cells that produce TGF-*β* also have TGF-*β* receptors, and therefore can perform autocrine signaling. Cancer cells increase their production of TGF-*β*, which affects the cells around them. Newly discoveries in the field of tissue engineering try to reestablish tissues injured by to pathologies, trauma, or congenital defects. Large mandibular or maxillary bone defects due to trauma or residual of large tumour removal have been commonly treated by using autologous graft [12]. Three-dimensional maxillary bone volume recoveries choosing autogenous bone graft is today a stable and predictable treatment option. On the other hand, it has been underlined how the bone collected from the same patient's body areas is usually grafted with postoperative difficulties, biological damages, irritation, or pain at the bone-grafting zone [13–18]. Nowadays, the biomaterials of use for facial bone reconstructions are many and of different derivation; moreover, thanks to the digital; it is possible to calculate the quantity of biomaterial needed or obtain the printing of the biomaterial with the ideal shape [19–25].

In the last 20 years, documented conventional guided bone regeneration procedures have been enhanced rebuilding vertical and horizontal maxillary bone defects guaranteeing to the patients' final prosthetic rehabilitation with functions and aesthetics. However, the time for having the final restoration is usually long, and at the same time, the presence of the scar tissue and the consequent no good healing of the soft closure tissues are a clinical condition that often happens. For this reason, recent investigations have been performed on the use of growth factors. Encouraging results have been obtained in clinical studies which are aimed at achieving successful outcomes in regenerative medicine and surgery, reducing the scar tissue in the soft-tissue healing, and promoting a quick healing in the regenerative surgery. The connection between growth factor and scaffold has been performed in order to limit the known disadvantages of the growth factor clinical applications [26].

Accordingly, to the National Science Foundation workshop in 1988, the term “tissue engineering” was officially established in order to mean the application of principles and methods of engineering and life sciences toward the fundamental understanding of structure-function relationships in normal and pathological mammalian tissues and the development of biological substitutes to restore, maintain, or improve tissue function [13, 27–30].

### 1.2. Aims

In this revision, authors will discuss the different therapeutic option in the field of reconstructive craniofacial treatment using scaffold and growth factors. The present revision is aimed at overviewing the most recent literature based on therapeutic and experimental possibilities of new scaffold biomaterials applied in the oral and maxillofacial surgery [31].

Moreover, author's purpose is to examine manuscripts about growth factors and relative scaffold applied for facial bone reconstruction in order to determine safe recommendations regarding the opportunity of substituting autologous bone graft for jaw atrophic reconstruction defects [32–36].

- What is the influence of growth factors use in oral reconstructive surgery to dental patients with bone defects compared to conventional surgery techniques?

## 2. Results

### 2.1. Study Selection

**Figure 1:**
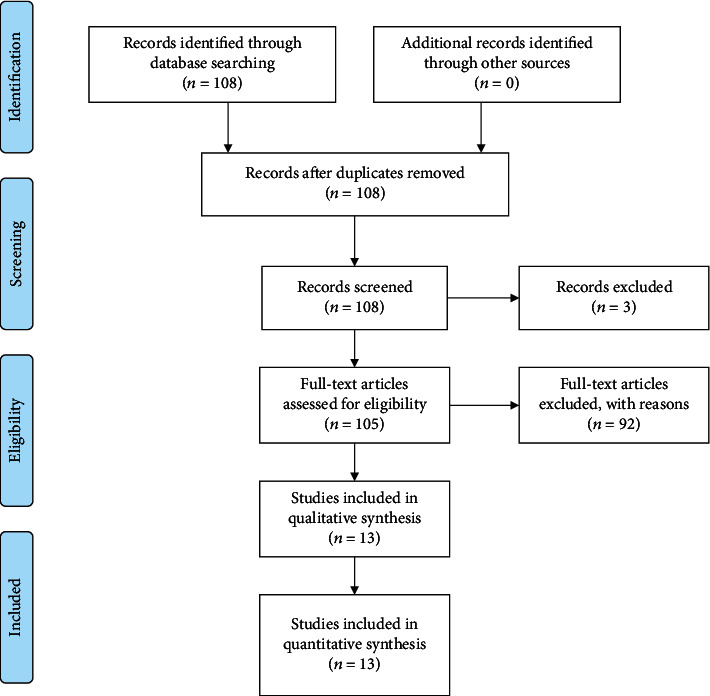
PRISMA flow chart.

From 108 results initially, then 20-year screening was carried out obtaining 105 results and, subsequently, only the relevant manuscript types were evaluated, in accordance with the Materials and Methods. At this point, 13 results have been obtained (Figure 1).

### 2.2. Risk of Bias within Studies

Risk of bias within studies has been evaluated for included RCT (radomized clinical trial) and showed in Table 1.

### 2.3. Results of Individual Study

### 2.4. Synthesis of Results

Thoma et al. [37] evaluated differences between xenogeneic block loaded with rhBMP-2 vs. autogenous bone blocks. Authors measured horizontal ridge width prior, after, and at 4 months after surgery. PROMs (patient-reported outcome measures) have been reported, and an additional analysis (histological examination) has been performed too. A higher morbidity was reported in the control group during surgery, and the biopsy revealed better results about mineralized tissue in the control group; but there are no significant differences between groups on horizontal width. Huang et al. [38] evaluated the effect of concentrated growth factor during GBR (guided bone regeneration). They performed a cone beam computed tomography (CBCT) evaluating the bone resorption rate and the bone density improvement with better results in CGF group than in ADM (acellular dermal matrix) group. In the Dragonas et al. [42] case report, they showed a GBR with the use of rhBMP-2 combined with xeno- and allografts and a 5-month dental implant placement in a patient. After implant surgery, they evaluated dehiscence on all inserted implants and then performed dental implant removal and a second GBR without the use of rhBMP-2 but only with the use of a xenograft. Chiantella [43] showed outcomes of a horizontal ridge augmentation with the use of rhPDGF-BB mixed with deproteinized bovine bone and covered with a porcine collagen membrane hydrated with the same growth factor. This surgery was conducted due to the premature loss of a maxillary central incisor with the complete dehiscence of the buccal plate. Santana and Santana [39] evaluated the differences between GBR performed with rhPDGF-BB in a (*β*-TCP)/hydroxyapatite (HA) carrier vs. autogenous bone reconstruction. They after surgical site exposure performed intramarrow penetration and filled defect with biomaterials. For experimental (GBR) group, they used a nonabsorbable barrier membrane. Authors performed bone measurements with a calibrated caliper at 1 mm below the highest point of the crest in the implant placement site (using an acrylic resin stent). Measurements were repeated at 6 months. FIT (final implant insertion torque) was evaluated too during the implant surgery, and implant placing was followed by an additional grafting if needed. There were no statistically differences between groups and follow-up time. Chiang et al. [44] used a modified ridge split augmentation with the use of rhPDGF-BB. After CBCT examination and local and systemic antiseptic prophylaxis, they exposed the bone defect under local anesthetic administration. Piezoelectric surgery was used to perform the crestal and vertical bony incisions (5 to 8 mm subcrestal). After corticotomies, ridge expansion was performed with the intraosseous application of FDBA hydrated with water and rhPDGF-BB. At the end, they used a resorbable collagen membrane. Bone width measurements were performed before surgery and 6 months after surgery, before dental implant placement. Amorfini et al. [40] in their RCT evaluated the differences in bone volume and stability between GBR with or without the use of growth factor (rhPDGF-BB) in mandibular atrophic ridges. RCT was conducted using a parallel and split mouth model. Bone graft intervention consisted of bone chips collected with a scraper and mixed with DBB (deproteinized bovine bone) covered with a resorbable membrane with or without the use of rhPDGF-BB. There were no statistically differences between groups in bone volume, neither at 1 year of follow-up. Urban et al. [45] conducted a study reporting the use of rhPDGF-BB in posterior maxillary area. In this case report, the authors specified the use of anorganic bovine bone infused in rhPDGF-BB. They used a sized collagen membrane and titanium pins too. In this thick biotype patient, they observed a horizontal bone increase at 9 months. Sclar and Best [46] conducted a GBR with the use of rhBMP-2 and bovine bone. They inserted a dental implant at 14 weeks from surgery. Guze et al. [47] evaluated the effect of a GBR with rhPDGF-BB in cancellous freeze-dried bone mineralized allograft with titanium mesh. Patient was examined at 1, 2, 4, 8, 12, and 24 weeks after surgery. Vertical and horizontal ridge measurements were performed, and a bone biopsy was conducted with a trephine bur. They showed a horizontal and vertical ridge augmentation. Urban et al. [48] conducted a GBR with the use of rhPDGF-BB with autogenous bone and a titanium reinforced e-PTFE (expanded poly-tetrafluoroethylene membrane) membrane. Simion et al. [49] evaluated the use of autogenous bone graft and deproteinized bovine bone particles hydrolyzed with rhPDGF-BB. Jung et al. [41] evaluated the effect of rhBMP-2 on GBR techniques. The use of xenogenic bone and collagen membrane could be improved by rhBMP-2. They placed 34 dental implants requiring lateral ridge augmentation due to a bone defect. The test group is represented by xenogenic bone substitute in addition with rhBMP-2. They evaluated defect height and conducted a histomorphometric analysis, with mineralized bone and surface of the bone in contact with newly formed bone.

## 3. Discussion

### 3.1. Summary of Evidence

Regenerative medicine now represents a therapeutic reality applicable to various organic substrates, which is aimed at repairing deficient tissues and restoring normal organ function. Among the possible specialist uses, in the dental field, the treatment of periodontal bone defects should be mentioned. These methods have also found space in the regeneration of peri-implant defects. The techniques currently in use involve the use of different materials. Among the various molecules, the group of fibroblast growth factor (FGF) is mentioned here, with particular interest in type 2. FGF was discovered in 1974, within the pituitary gland of the bovine, as a factor capable to stimulate the proliferation of fibroblasts. Ten years later, two proteins (FGF-1 and -2) were distinguished on the basis of the degree of acidity-basicity. Over the next two years, the amino acid sequence of bovine FGF-2 was identified and the corresponding human cDNA was cloned. Other genes encoding FGF were searched by analogy: to date, 22 have been identified. The proteins have been classified into 7 subfamilies on the basis of the amino acid structure: the first of these includes the 2 original molecules, FGF-1 and -2. Each polypeptide consists of 150-200 amino acid residues, with a core characterized by high levels of homology. As far as signal transmission is concerned, 4 different receptors are classified (7 net of alternative splicing processes), all with tyrosine kinase activity. The different FGFs play an important role in the differentiation and growth processes of different embryonic cytotypes, including human odontogenesis. They also have an action in tissue repair and regeneration mechanisms. FGF-2, which anticipated as the molecule of greatest periodontal interest, manifests proliferative signaling activity of blood vessels/capillaries [50]: in the medical field, it finds application in the treatment of complex wounds and mesenchymal cells. It appears that the molecule also plays a role in the differentiation of these cellular precursors to osteoblasts. As regards the use in tissue engineering of the periodontium, FGF-2 seems to have a control activity on the differentiation of the cells of the periodontal ligament and, at the same time, stimulates cell proliferation. Clinical trials, recently confirmed by a systematic review, affirm its efficacy in the regeneration of periodontal bone defects. Thoma et al. [37] concluded that both treatment modalities were successful in regenerating bone, despite there were more mineralized tissues in the autogenous block group pain during surgery favour the test group. Huang et al. [38] concluded that the use of CGF derived by venous blood by centrifugation could be recommended to patients with alveolar cleft as a better performing therapeutic strategy. Dragonas et al. [42] affirmed that direct conclusions regarding the positive or negative effects of rhBMP-2 in bone augmentation cannot be made, but based on the ridge resorption using this approach, the decision was made to complete the second ridge augmentation using a different regenerative approach, using only xenograft, which has been shown to maintain augmentation dimensions predictably over time. Authors demonstrated in detail a complication associated with rapid resorption of regenerated bone following ridge augmentation using rhBMP-2 in combination with allograft and xenograft. According to Chiatella [43], the use of rhPDGF-BB and collagen membrane provides a satisfactory soft-tissue healing and a bone tissue stability at 2 years. According to the author, further clinical studies are necessary to evaluate these conditions and therapies. Santana and Santana [39] concluded that growth factor could enhance synthetic material properties, providing similar results to autogenous block. Chiang et al. [44] demonstrated that their ridge splitting technique had a low morbidity with significant horizontal bone gain; thanks to the use of growth factors. Amorfini et al. [40] concluded that in GBR, the block allograft and the standard regenerative procedure showed similar results, but they underline that rhPDGF-BB positively influenced the soft-tissue healing. Urban et al. [45] affirmed that this treatment modality could eliminate the need for bone harvesting. Sclar and Best [46] concluded that further studies are necessary to evaluate the potential of growth factor. Guze et al. [47] concluded that the combination of FDBA, rhPDGF-BB, and titanium mesh could provide minimally invasive alternative for severe resorbed alveolar ridge. Urban et al. [48] in their study concluded that growth factor could eliminate completely the need of bone harvesting. Simion et al. [49] concluded that this surgery, which is aimed at placing dental implant, was successful. Jung et al. [41] concluded that rhBMP-2 could enhance the maturation process and could increase the graft to bone contact in humans.

In implantology, it is essential to know and evaluate the quantity and quality of bone available. Often clinicians have to intervene in situations of “bone atrophy,” that is, in cases of reduced alveolar bone volume, due to a previous loss of teeth (edentulous) or due to the destructive effect of pyorrhea (periodontitis). In order to ensure effective implant-prosthetic rehabilitation, the research has developed several protocols that use autologous, heterologous, or mixed materials. Science attests that the addition of certain growth factors such as rhPDGF or platelet rich plasma (PRP) inside the alveolus is able to increase the local concentration of growth factors, reducing the time required for complete bone regeneration and producing a denser bone. The autologous platelet-rich plasma, thanks to the abundant presence of growth factors, represents a valid aid for the acceleration of the repair processes and the regeneration of hard and soft tissues, in oral surgery [51–53].

The search for products similar to PRP that contain a greater number of growth factors is constantly evolving. Over the years, numerous implementation protocols have been proposed for the PRP. To date, technology has made it possible, by following the principles of PRP, to obtain concentrates such as CGF (concentrated growth factors). CGF is a valid aid in speeding up the processes of bone and soft-tissue regeneration. Its use has been proposed in various situations ranging from filling postextraction alveoli to filling cavities after cystectomies or in breast enhancement. In order to speed up osteoinductive activity, some specialists suggest wetting the surface of the implants with CGF [54–57]. The latter can be used alone or together with autologous particulate bone or biomaterials. Although the literature is still uncertain on the clinical protocols for the use of platelet concentrate, to date, this tool seems to be a valid aid in the bone repair process and, considering the great role that growth factors play on cells, the use of the concentrate will become without other more relevant than other preparations. Thanks to its great regenerating properties; the PRP treatment by stimulating cell proliferation, angiogenesis, and revascularization of the tissues represents the most advanced alternative to obtain exceptional results in a short time and surprisingly effective [58–62].

Among the new methods used to increase tissue regenerative potential, in the last decade, the introduction of platelet-rich plasma (PRP), a platelet concentrates rich in growth factors (GFs), whose use would seem to determine an increased speed of bone and mucosal healing [38, 63–65].

Although it has been shown that individual growth factor stimulates the proliferation of cell lines involved in the healing process (bone stromal cells, osteoblasts, and fibroblasts), there is still little evidence about the interaction modalities of all the signal molecules released in the surgical site after application of PRP [66–68].

### 3.2. Limitations

Unfortunately, it is necessary to mention some limitations of this study, the first of all being the small number of RCTs in this area. Furthermore, given the great variability of outcomes, it is not possible to carry out a single statistic or a forest plot. The inclusion of case reports can lead to a risk of bias by not often having control groups.

## 4. Materials and Methods

### 4.1. Protocol and Registration

The systematic review was conducted in accordance with the PRISMA (Preferred Reporting Items for Systematic Reviews and Meta-Analyses) protocol; the template and guidelines in fact follow these guidelines. In addition, the systematic review was recorded on the University of York website, PROSPERO (international prospective register of systematic reviews), on 197445 and with the number 07/07/20. This review used a PICO (population, intervention, comparison, and outcome) question to state the aim of the study.

### 4.2. Eligibility Criteria

The following criteria were used to filter the results obtained during electronic research:

Inclusion Criteria

- RCT or clinical trial or case report about growth factor use in oral bone regeneration
- Human studies

Exclusion criteria

(i)Older than 20 years publication

(ii)Not in English manuscripts

(iii)Not accessible abstract or title

(iv)PhD thesis or letter or editorials

### 4.3. Information Sources

The sources of information taken into consideration for this study are the scientific search engines PubMed Embase, and Scopus Elsevier; in addition, a manual search was carried out in the textbooks relating to the topic.

### 4.4. Search

The research was conducted once the search criteria were identified. The keywords have been scientifically evaluated and criticized by the different authors, in order to obtain the highest possible number of results and limit the risk of bias. Once an agreement was found on the keywords, the search was carried out in the search engines listed in the previous paragraph on 01/05/2020. The keywords used are as follows: ““guided bone regeneration “AND” growth factor””.

### 4.5. Study Selection

The selection of the results from the research was carried out according to the parameters listed in the previous paragraphs. Following the application of digital filters, provided by the search engines, manual screenings were carried out regarding the reading of titles and abstracts in order to identify articles not in accordance with the selection criteria. Subsequently, reading of the full text for the inclusion or otherwise of the individual result was performed.

### 4.6. Data Collection Process

During the reading of the full text of the articles included, data were collected. The individual data were obtained from Materials and Methods and Results of the individual article; the latter were analyzed and then used in this systematic review.

### 4.7. Data Items

Data items have been defined by authors, and they have been used as follows in the tables.

(i) Table 1 (according to Cochrane risk of bias [69–71])
  - *Author*: this includes the first author name and year of the manuscript publication
  - *Random Sequence Generation* (*Selection Bias*): sample sequence generation
  - *Allocation Concealment* (*Selection Bias*): randomized allocation concealment in groups
  - *Blinding of Participants and Personnel* (*Performance Bias*): participant blinding
  - *Blinding of Outcome Assessment* (*Detection Bias*): blinded outcomes to operator
  - *Incomplete Outcome Data* (*Attrition Bias*): missing data
  - *Selective Reporting* (*Reporting Bias*): selective data showing
(ii) Table 2
  - *Authors and Year*: these include the first author name and year of the manuscript publication
  - *Type of Study*: type of article (RCT or case report only)
  - *Groups*: type of groups for RCT or used methods for case report
  - *Outcomes*: evaluated outcomes from the study
  - *Main Results*: brief numerical results obtained from the study analysis
  - *Statistic*: statistical results of the study
(iii) Table 3
  - *Measure*: summary of the results obtained outcomes

### 4.8. Risk of Bias in Individual Study

Risk of bias examination has been conducted according to Cochrane guidelines on obtained results [69–71]. A bias is a systematic error or deviation from the truth, in results or inferences. Biases can operate in either direction.

### 4.9. Summary Measures

All included studies were analyzed by authors, and evaluated outcomes have been shown in Table 3.

### 4.10. Synthesis of Results

The summary of the results was carried out manually by the authors of the manuscript, especially once carried out at the manual synthesis of the results obtained by the individual article; this was revised by all the authors.

## 5. Conclusions

All the results analyzed, although not in conformity with each other, as regards materials, methods, and results, follow a common guideline. In fact, all the results obtained are in agreement to show an improvement in the clinical conditions with the use of growth factors. In particular, growth factors can improve surgical outcomes, both related to the operating field (improved height and bone thickness) compared to conventional techniques (without the use of growth factors) and to the patient's systemic field (improving the quality of life, postoperative phases, and self-reported measures by the patient). Certainly, further studies are needed to analyze in more detail the differences between the different growth factors and their performance.

## Figures and Tables

**Table 1:** Risk of bias of individual RCT (radomized clinical trial) study according to Cochrane.

Author	Random sequence generation (selection bias)	Allocation concealment (selection bias)	Blinding of participants and personnel (performance bias)	Blinding of outcome assessment (detection bias)	Incomplete outcome data (attrition bias)	Selective reporting (reporting bias)
Thoma et al. [37] 2018	+	+	-	-	+	+
Huang et al. [38] 2018	+	+	-	-	+	+
Santana and Santana [39] 2015	+	+	-	-	+	+
Amorfini et al. [40] 2013	+	+	-	-	+	+
Jung et al. [41] 2003	-	+	-	-	+	+

**Table 2:** Results of individual study. This table shows numerical results of individual study, and it is formatted according to Materials and Methods.

Author and year	Type of study	Groups	Outcomes	Main results	Statistic
Thoma et al. [37] 2018	RCT; 24 patients	Xenogenic bone block loaded with rhBMP-2 (test) vs. autogenous bone block (control)	Horizontal ridge width, patient-reported outcome measures (PROMs), histologic examination	Median ridge width increased from 4.0 mm (Q1 = 2.0; Q3 = 4.0) (test) and 2.0 mm (Q1 = 2.0; Q3 = 3.0) (control) to 7.0 mm (Q1 = 6.0; Q3 = 8.0) (test) and 7.0 mm (Q1 = 6.0; Q3 = 8.0) (control) at 4 months.	*P* > .05
Huang et al. [38] 2018	RCT; 20 patients	GBR (guided bone regeneration) using acellular dermal matrix (ADM) film combined with alveolar bone grafting vs. alveolar bone grafting combined with concentrated growth factor (CG)	Bone resorption rate, bone density	Bone density improvement in CGF (concentrated growth factors) (61.62 ± 4.728%) and in cases with ADM (27.05 ± 5.607%)	Bone density *P* = 0.0002
Dragonas et al. [42] 2017	Case report	Bilateral maxillary sinus and ridge augmentation procedures using rhBMP-2 combined with allograft and xenograft	After surgery complication (dental implant dehiscence)	Dental implant insertion torque >25 N/cm	/
Chiantella [43] 2016	Case report	Horizontal ridge augmentation surgery with deproteinized bovine bone xenograft particles combined with rhPDGF-BB with collagen membrane	Soft-tissue healing; bone tissue quality and stability; peri-implant tissue stability	The torque insertion of after surgery implants exceeded 55 N/cm	/
Santana and Santana [39] 2015	RCT; 30 patients	rhPDGF-BB GBR with beta-tricalcium phosphate (*β*-TCP)/hydroxyapatite (HA) as carrier vs. autogenous bone block	Bone crest width (BCW); final implant insertion torque (FIT)	Mean baseline BCW measurements were 3.13 ± 0.9 mm for the control group and 3.03 ± 0.7 mm for the experimental group. Implant was placed with torque values > 35 N/cm in 93% of control sites and 87% of experimental sites.	BCW (*P* = .733); FIT (*P* = .32).
Chiang et al. [44] 2014	Case report; three patients	Modified ridge split with rhPDGF-BB hydrated crushed cortical freeze-dried bone allograft and collagen membrane using	BCW; cone beam computed tomography (CBCT) evaluation	All cases showed more than 5 mm of BCW gain.	/
Amorfini et al. [40] 2013	RCT; 16 patients, 50 dental implants	Deproteinized bovine bone added to autologous bone vs. deproteinized bovine bone added to autologous bone with rhPDGF-BB	Quantity of bone variation at 1 year	From 0.19 cm^3^ to 0.18 cm^3^ at 1 year for GBR and from 0.19 cm^3^ to 0.16 cm^3^ for allograft	*P* = .25
Urban et al. [45] 2013	Case report	rhPDGF-BB in conjunction with autogenous bone and anorganic bovine-derived bone mineral and a barrier membrane to reconstruct a severe alveolar posterior maxillary bone defect	Postoperative discomfort; bone width	6 mm bone width augmentation	/
Sclar and Best [46] 2013	Case report	rhBMP-2	Tissue stability	Peri-implant tissue was stable, and surgery was uneventful.	/
Guze et al. [47] 2013	Case report	rhPDGF-BB in conjunction with an overlying titanium mesh in posterior mandibular ridge defect	CT scan; histologic and micrographic analysis	10 mm increase in ridge height as measured from the dense cortical mature bone to the crest of the augmented bone. A number of residual allograft particles were seen surrounded by newly formed bone.	/
Urban et al. [48]	Case report	Autogenous bone in conjunction with rhPDGF-BB and barrier membrane	Bone height	About 2 mm of exposed root surface was in contact with bone.	/
Simion et al. [49] 2008	Case report	1 : 1 ratio of autogenous bone graft and deproteinized bovine bone particles with rhPDGF-BB	/	/	/
Jung et al. [41] 2003	RCT; 11 patients, 34 dental implants	Xenogenic bone substitute and collagen membrane coated with rhBMP-2 and implant insertion (test); xenogenic bone substitute and collagen membrane and implant insertion (control)	Peri-implant bone defect height; histology	Peri-implant bone defect means were 7 mm and 5.8 in at test and control baseline, respectively. At reentry, the mean decreased to 0.02 for test and 0.04 for control. Histomorphometric analysis showed an average area density of 37% (SD 11.2, range 23-51%) newly formed bone at test sites and 30% (SD 8.9, range 18-43%) at control sites.	Peri-implant bone defect *P* < 0.01

**Table 3:** Summary measures. This table shows all evaluated outcomes by single results.

Measures
Horizontal ridge width, patient-reported outcome measures (PROMs), histologic examination, after surgery complication, bone resorption rate, bone density, soft-tissue healing; bone tissue quality and stability; peri-implant tissue stability, bone crest width (BCW); final implant insertion torque (FIT), micrographic analysis, peri-implant bone defect height
